# Framing the Unlikely Pair: “Free Gifts” Heuristic in Evaluation of Non-Complementary Product Bundles

**DOI:** 10.3390/bs15091168

**Published:** 2025-08-27

**Authors:** Zhiying Li, Seunghee Han

**Affiliations:** Department of Business Administration, Chung-Ang University, Seoul 06974, Republic of Korea; jiyeong23@cau.ac.kr

**Keywords:** product bundling, complementarity, promotional framing, smart shopper feelings, consumer psychology, decision-making

## Abstract

Firms increasingly offer non-complementary product bundles, yet marketing these ‘unlikely pairs’ effectively remains a significant challenge. This study investigates a solution by examining the psychological impact of promotional framing. Using a 2 × 2 between-subject online experiment (*N* = 342), we test how a ‘free gift’ versus a ‘price discount’ frame moderates the effect of bundle complementarity on purchase intention. Our findings reveal that while non-complementary bundles typically reduce consumers’ smart shopper feelings and purchase intentions, this negative effect is pronounced under a price discount frame but attenuated under a free gift frame. Specifically, the free gift framing prevented the decline in smart shopper feelings observed in the discount condition, resulting in non-complementary bundles being evaluated as favorably as complementary ones. These findings indicate that the challenge of marketing non-complementary bundles can be mitigated by using a free gift frame, which leverages a powerful psychological heuristic to sustain consumers’ feelings of savvy consumption and enhance the appeal of otherwise incongruent offers.

## 1. Introduction

Product bundling is a long-standing marketing strategy designed to enhance perceived value and drive consumer purchases. Yet, its success is not guaranteed. As creative but seemingly mismatched bundles—such as a coffee maker paired with a novel or a software suite with an unrelated online course—become increasingly common, a critical question in consumer psychology emerges: what makes these non-complementary offers effective?

Research on product bundling has demonstrated that consumer evaluations are shaped by the degree of complementarity between the bundled components. Many studies suggest that bundles with high complementarity (e.g., a printer with an ink cartridge) are intuitively appealing and often increase profitability compared to selling items separately (Leszczyc & Häubl, 2010). This preference is typically attributed to the functional synergy and added utility consumers derive from joint consumption. However, evidence also indicates that non-complementary bundles are not invariably disadvantaged. Depending on the context, they can be equally, or even more, attractive than complementary bundles. For example, Karataş and Gürhan-Canli (2020) found that consumers with an abstract mindset favored non-complementary bundles over complementary ones. These findings suggest that consumer preferences for bundle types are more nuanced than a simple hierarchy of complementarity and highlight the importance of identifying the psychological conditions under which non-complementary bundles can succeed.

Consumers consistently strive for value-maximizing consumption, a phenomenon often characterized as “smart shopping.” This behavior involves investing effort in collecting information, comparing products, and making choices that affirm one’s efficacy as a consumer. Identifying the psychological factors that evoke such “smart shopper feelings” represents a valuable avenue for understanding economic decision-making. Complementary bundles often elicit these feelings because their combined value is intuitively clear. Yet the persistence and popularity of non-complementary bundles in the marketplace challenge the notion that complementarity is always paramount. Their success suggests that consumers’ evaluations are not determined solely by functional synergy but also by other perceived benefits, such as the distinctiveness or uniqueness of the offer. While consumer mindset offers one explanation for these evaluations, promotional framing may represent a more direct and externally controllable factor. Prior research has robustly shown that consumers often prefer receiving a ‘free gift’ over a financially equivalent price discount (e.g., Chandran & Morwitz, 2006; Kamins et al., 2009; Roll & Pfeiffer, 2017), perceiving it as a distinct psychological gain rather than a mere reduction in loss consistent with principles of prospect theory. However, this principle has not been systematically examined in the context of bundle complementarity. Thus, it remains unclear whether and how promotional framing can compensate for the lower intuitive value of non-complementary bundles. This study addresses this gap by investigating the interaction between promotional framing (free gift vs. price discount) and bundle complementarity (complementary vs. non-complementary) on purchase intentions, mediated by ‘smart shopper feelings.’ We propose that the psychological benefit of a ‘free gift’ frame is powerful enough to mitigate the perceived incongruity of non-complementary bundles, making them as appealing as their complementary counterparts. By contrast, we expect the effectiveness of a standard price discount frame to depend more heavily on the intuitive logic of a complementary bundle. By examining these dynamics, our research clarifies when and why non-complementary bundles can be effective, offering both theoretical insights into consumer value perception and practical guidance for designing more innovative bundled offers. The rest of this article is structured as follows: First, prior research on bundle complementarity, smart shopper feelings, and promotion framing is reviewed to establish hypotheses. Next, these hypotheses are tested through experiments. Finally, the theoretical and practical implications of the research findings are discussed.

## 2. Theoretical Background and Hypotheses Development

### 2.1. Bundle Evaluation: The Central Role of Complementarity

Product bundling, the practice of selling two or more products in a single package, is a foundational marketing strategy (Stremersch & Tellis, 2002). The success of a bundle hinges on the consumer’s perception of its value, which is critically influenced by how the supplementary items relate to the core product (Gaeth et al., 1991; Yadav, 1994). A primary driver of this value perception is the complementarity between the bundled components—defined as the degree to which the products enhance each other’s utility when consumed together (Guiltinan, 1987). By contrast, non-complementary bundles consist of products that provide distinct, independent sources of utility without directly enhancing one another’s value (Yadav, 1994; Karataş & Gürhan-Canli, 2020).

From a consumer psychology perspective, complementary bundles are often more effective because they are easier to process and justify. They possess high cognitive fluency; a coffee machine bundled with compatible coffee pods, for instance, creates an intuitive and easily understood narrative of use. This ease of processing enhances the perceived value and attractiveness of the offer (Labroo & Pocheptsova, 2016). Furthermore, complementary items can generate synergistic value, where the joint consumption experience is perceived as greater than the sum of its parts. A fitness tracker paired with a premium subscription to a health app, for example, offers a more holistic and enhanced wellness experience, boosting the bundle’s overall utility.

Conversely, bundles with low complementarity demand greater cognitive effort from consumers. An offer combining a primary product (e.g., a laptop) with a functionally unrelated item (e.g., a subscription to a music streaming service) requires the consumer to construct separate justifications for the purchase. This can diminish the perceived coherence and value of the deal, introducing cognitive dissonance. The literature has consistently shown that bundling is most profitable when products are complements and less effective when they are substitutes or unrelated (Leszczyc & Häubl, 2010; Roach, 2011).

Therefore, to establish a foundational baseline for our investigation, we first hypothesize that a lack of complementarity will reduce consumers’ purchase intention:

**H1.**  *A lack of complementarity in bundled products will decrease consumers’ purchase intention.*

### 2.2. The “Smart Shopper Feeling” as a Mediator

While the economic utility of bundles is well-established (Venkatesh & Kamakura, 2003), a bundle’s success also depends on its ability to generate positive affective responses. A key affective response is the “smart shopper feeling”—the sense of pride or accomplishment a consumer experience when they believe they have secured an especially good deal (Schindler, 1989). This feeling goes beyond the monetary savings themselves; it is a self-relevant emotion that validates consumers’ effort, competence, and savviness as shoppers. In this sense, the act of “outsmarting” the system provides an ego-expressive benefit that strengthen consumers’ self-concept (Schindler, 1992; Zhang & Mick, 2019).^1^

We propose that bundle complementarity is a direct antecedent of this feeling. A complementary bundle (e.g., a gaming console with a perfectly matched controller) presents a coherent and synergistic offer that is easy to process and justify. Prior research shows that such fluency in evaluation enhances perceived value (Labroo & Pocheptsova, 2016) and strengthens the consumer’s confidence in their choice (Darke & Chung, 2005). By recognizing and selecting this logical package, consumers can more easily affirm their own good judgment, which in turn fosters the smart shopper feeling. In contrast, a non-complementary bundle lacks this intuitive fit, introducing ambiguity that undermines decision confidence (Dhar & Sherman, 1996) and thereby dampens the experience of feeling like a savvy shopper.

This affective response, or lack thereof, should in turn directly influence willingness to purchase. When consumers feel like smart shoppers, they view their choice more favorably, increasing purchase intention. This leads to the proposition that smart shopper feelings area psychological mechanism through which bundle complementarity affects purchase intentions. Therefore, we hypothesize the following:

**H2.**  *Smart shopper feelings will mediate the relationship between bundle complementarity and purchase intention, such that a lack of complementarity will lead to weaker smart shopper feelings, which in turn will decrease the intention to purchase the bundle.*

### 2.3. The Power of Framing: Why a “Free Gift” Outweighs a Discount

Beyond a bundle’s composition, its perceived value is profoundly influenced by how the promotion is framed (e.g., Ding & Zhang, 2020; Lee & Yi, 2019; Song et al., 2023; Yadav & Monroe, 1993). This study focuses on a fundamental and powerful distinction: framing a promotion as a monetized price discount versus a non-monetized free gift. We argue this choice of frame is a critical factor that interacts with bundle complementarity to shape consumer perceptions.

The psychological allure of “free” is well-documented. Consumers consistently exhibit a strong preference for receiving a free gift over an economically equivalent price discount (Chandran & Morwitz, 2006; Roll & Pfeiffer, 2017). This preference is best explained through the lens of mental accounting (Thaler, 1985). A price discount is typically integrated with the purchase cost and processed as a reduced expense, whereas a free gift is mentally segregated into a separate “account” and experienced as an additional gain. Because the gain is coded as distinct, it generates higher transaction utility—the psychological satisfaction derived from securing an advantageous deal (Diamond & Campbell, 1989).

Although prior work has identified a “freebie devaluation effect,” where items may be discounted in quality when offered for free (Raghubir, 2004), this effect is often attenuated in more complex evaluations such as bundles. Kamins et al. (2009) argue that when consumers face a more complex evaluation, the increased cognitive load leads them to rely more on simple, powerful cues or heuristics. In this context, the positive and unambiguous heuristic of a “free” gain tends to overpower the more complex, negative inferences required for devaluation.

### 2.4. Interaction Effects and Hypotheses

Based on this theoretical foundation, we expect the impact of promotional framing to depend on the bundle’s complementarity.

For a complementary bundle, the offer is already logical and easy to evaluate. Both a price discount and a free gift frame should be effective, as they both enhance an already appealing package. This cognitive fluency should foster smart shopper feelings and boost purchase intentions under either frame.

The crucial difference emerges with a non-complementary bundle. When framed as a price discount, the offer highlights the overall expense of a bundle whose components do not naturally fit together. This lack of synergy undermines the sense of transaction utility, resulting in lower smart shopper feelings. In contrast, when the same bundle is framed as including a free gift, the negative impact of non-complementarity is attenuated. Because the free gift is mentally segregated into a separate account and experienced as a distinct gain (Thaler, 1985), it provides a positive focal point that offsets the bundle’s lack of coherence. As a result, smart shopper feelings for non-complementary bundles rise to a level comparable to those for complementary bundles. This reasoning leads to our central interaction hypotheses:

**H3.**  *Promotion framing will moderate the effect of bundle complementarity on smart shopper feelings, such that the effect of complementarity will be significant under a price discount frame but will be attenuated under a free gift frame.*

**H4.**  *This interaction will be carried through to purchase intention via smart shopper feelings. Specifically, promotion framing and bundle complementarity will interact to influence smart shopper feelings, which in turn will influence purchase intention (i.e., a moderated mediation model).*

**Figure 1:**
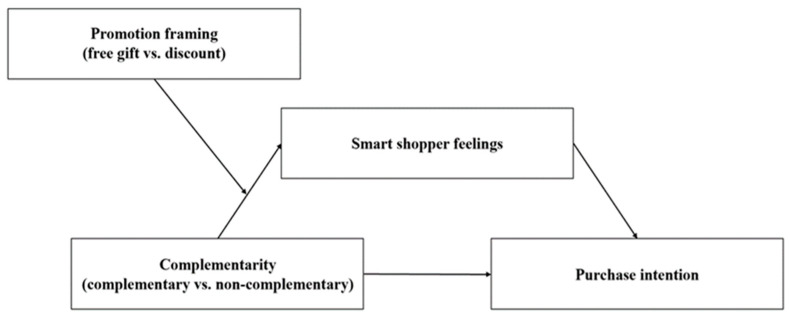
Conceptual model of moderated mediation.

Figure 1 illustrates the proposed theoretical framework. Bundle complementarity (the independent variable) is predicted to influence purchase intention (the dependent variable) both directly (H1) and indirectly through the mediator, smart shopper feelings (H2). This indirect effect is hypothesized to be moderated by promotion framing (H3), such that the path from bundle complementarity to smart shopper feelings is weaker under a free gift frame. H4 proposes the overall moderated mediation model.

## 3. Materials and Methods

### 3.1. Overview of the Study

This study employed a 2 (bundle complementarity: complementary vs. non-complementary) × 2 (promotion framing: free gift vs. discount) between-subject experimental design to test the proposed hypotheses.

To create a realistic and engaging scenario for participants, a hypothetical cruise package was selected as the context for the experiment. This context was chosen because premium, experience-based purchases are well-suited for examining the interplay between cognitive and affective factors in decision-making. The evaluation of such experiential products often involves complex trade-offs, making it a fertile ground for testing how consumers respond to different value propositions and framing effects.

The study was designed to test four key hypotheses: that complementary bundles would be preferred over non-complementary ones (H1); that this effect would be mediated by smart shopper feelings (H2); and that this mediation would be moderated by promotion framing (H3 and H4), with the ‘free gift’ frame hypothesized to be particularly effective at increasing the appeal of non-complementary bundles.

### 3.2. Participants

Three hundred and sixty participants were recruited from Prolific in return for monetary compensation. Out of 360 responses from U.S. participants, we excluded 18 who failed a simple instructional attention check and used the data from the remaining 342 participants (Mage = 40.8 years; 43.6% female). Participants were randomly assigned to one of the four conditions created by crossing bundle complementarity (complementary vs. non-complementary) and promotion framing (free gift vs. discount).

### 3.3. Manipulations

To manipulate bundle complementarity, two types of promotional print advertisements were developed. In the complementary condition, participants were shown an advertisement that combined New York City (NYC) cruise tickets with a lunch experience on the cruise. This condition was considered complementary because the two services occur simultaneously and directly enhance one another’s value: enjoying a meal while taking in the scenery created a richer and more integrated consumption experience.

In the non-complementary condition, participants were presented with an advertisement combining NYC cruise tickets with a Times Square Wheel ticket. This combination was categorized as non-complementary because the two activities are temporally and experientially independent. Each offers distinct utility on its own, but they do not generate additional synergistic value when consumed together. Thus, the contrast between the two conditions reflects our conceptual definition of complementarity as the extent to which products provide synergistic versus independent value. To manipulate promotion framing, the type of promotion depicted in the advertisements was varied. In the free gift condition, the advertisement emphasized that a lunch experience or a Times Square Wheel ticket was provided at offered free, using the phrase “Now Free.” Conversely, in the price discount condition, the focus was on the reduction in the overall price of the bundle. The advertisement stated “Now you pay $70, Save $20!” to highlight the discount applied to the total bundle price. In the discount condition advertisements, the specific prices of each individual product within the bundle were intentionally omitted to emphasize the overall savings rather than the individual value of each component (For the full stimuli, see Appendix A).

To ensure a controlled comparison, the supplementary items were selected based on their identical market price. Both the on-cruise lunch (used in the complementary condition) and the Times Square Wheel ticket (used in the non-complementary condition) have a standard retail price of $20. This value was used to structure the price across all experimental conditions. In the price discount condition, the advertisement stated the total bundle price was “$70 (Save $20!),” reflecting the $90 combined value. In the free gift condition, the cruise ticket was priced at $70, and the supplementary $20 item was explicitly framed as “Now Free.” This design ensures that the final out-of-pocket cost to the consumer ($70) and the objective value of the supplementary item ($20) were held constant across all conditions. Therefore, any observed variations in purchase intention can be attributed directly to the manipulations of bundle complementarity and promotional framing, rather than underlying differences in price or value.

### 3.4. Measures

Purchase Intention: The dependent variable was measured using three items on a 7-point Likert scale (1 = “strongly disagree,” 7 = “strongly agree”). Participants rated their likelihood of buying the bundle, their happiness with the offer, and whether they found the bundle unattractive (reverse-coded). The measure demonstrated high internal consistency (α = 0.96).

Smart Shopper Feelings: The mediating variable was measured with a three-item, 7-point scale adapted from Schindler (1998). Participants rated the extent to which they would feel proud of their purchase, feel like a smart shopper, and feel good about themselves. This scale also proved highly reliable (α = 0.93).

Manipulation Check and Demographics: Following the main measures, a manipulation check assessed whether participants correctly perceived the bundle’s complementarity and the promotion type. Finally, participants provided demographic information.

## 4. Results

### 4.1. Manipulation Checks

To test the effectiveness of our bundle complementarity manipulation, we conducted an independent samples t-test, examining participants’ ratings of how complementary they viewed the bundles presented in the promotional print advertisements. Results from the *t*-test revealed a significant difference between the two experimental conditions. Participants in the complementary condition (M = 5.60, SD = 1.38) reported significantly higher perceptions of complementarity compared to those in the non-complementary condition (M = 4.90, SD = 1.58; t(340) = 4.50, *p* < 0.001).

In addition, to ensure the effectiveness of the promotion framing manipulation, we conducted independent samples *t*-tests, comparing participants’ ratings of whether the bundle included a free gift or was framed as a discount. Participants in the free gift promotion condition (M = 4.87, SD = 2.32) were significantly more likely to perceive the bundle as including a free gift compared to those in the discount promotion condition (M = 1.96, SD = 1.52; t(340) = 13.75, *p* < 0.001). Conversely, participants in the discount promotion condition (M = 6.43, SD = 1.06) rated the bundle as including a discount significantly higher compared to participants in the free gift promotion condition (M = 4.79, SD = 2.10; t(340) = −9.11, *p* < 0.001). Taken together, the results of these manipulation checks indicate that both the bundle complementarity and the promotion framing manipulations were successful.

### 4.2. Direct Effects on Purchase Intention

An independent samples *t*-test was conducted to examine differences in purchase intention for the bundle based on bundle complementarity. The results revealed a significant effect of bundle complementarity on purchase intention (t = 4.485, *p* < 0.001). Specifically, participants in the complementary condition (N = 170, M = 5.44, SD = 1.52) reported a significantly higher purchase intention compared to those in the non-complementary condition (N = 172, M = 4.65, SD = 1.75; t = 4.485, *p* < 0.001). These results suggest that the lack of complementarity in bundled products decreases consumers’ purchase intention, supporting Hypothesis 1.

### 4.3. Mediation Analysis: Smart Shopper Feelings

To test the prediction that smart shopper feelings would mediate the effect of bundle complementarity on purchase intention, we conducted a mediation analysis with PROCESS (SPSS, v5.0) Model 4 using 5,000 bootstrap samples (Hayes, 2022). Specifically, we examined the indirect effect of bundle complementarity (complementary vs. non-complementary) on purchase intention through smart shopper feelings. The mediation analysis revealed a significant indirect effect (B = −0.41, SE = 0.12, *p* < 0.001; 95% CI [−0.67, −0.13]). Specifically, participants in the non-complementary condition reported lower smart shopper feelings, which in turn decreased their purchase intention for the bundle compared to participants in the complementary condition, supporting Hypothesis 2.

### 4.4. Moderation Analysis: Promotion Framing

To examine whether promotion framing moderates the effect of bundle complementarity on smart shopper feelings, we conducted a two-way ANOVA with bundle complementarity (complementary vs. non-complementary), promotion framing (free gift vs. discount), and their interaction as the independent variables, and smart shopper feelings as the dependent variable. This analysis allowed us to test whether the impact of bundle complementarity on participants’ smart shopper feelings varied depending on the type of promotional framing presented.

The results revealed significant main effects of both bundle complementarity and promotion framing. Specifically, the main effect of bundle complementarity was significant (F(1, 338)= 9.21, *p* = 0.003, η*p*^2^ = 0.027), indicating that participants in the complementary bundle condition reported higher smart shopper feelings overall compared to those in the non-complementary bundle condition. The main effect of promotion framing was also significant (F(1, 338)= 6.59, *p* = 0.011, η*p*^2^ = 0.019), demonstrating that how the promotion was presented influenced participants’ smart shopper feelings.

More critically, these main effects were qualified by a significant interaction between bundle complementarity and promotion framing (F(1, 338)= 5.05, *p* = 0.025, η*p*^2^ = 0.015), indicating that the effect of bundle complementarity on smart shopper feelings was contingent upon the type of promotion framing used. This interaction suggests that the negative impact of non- complementarity on smart shopper feelings was not uniform across the different promotional framings but varied significantly based on how the promotional value was presented to participants.

**Figure 2:**
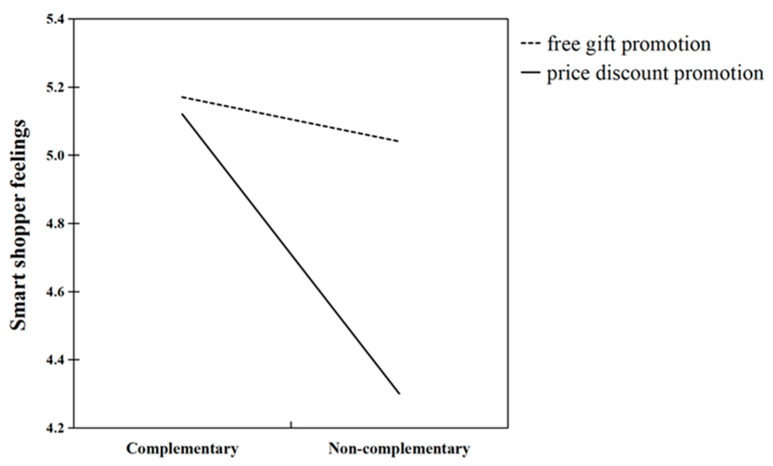
Promotion framing moderates the effect of bundle complementarity on smart shopper feelings.

As illustrated by Figure 2, this interaction was primarily driven by lower smart shopper feelings in the non-complementary condition when the bundle was framed as a discount (M_complementary_ = 5.12, M_non-complementary_ = 4.30, *p* < 0.001). In contrast, under the free gift frame, the difference between complementary and non-complementary bundles was non-significant (M_complementary_ = 5.16, M_non-complementary_ = 5.04, *p* = 0.561), suggesting that the free gift frame attenuated the negative effect of non-complementarity. Taken together, these findings indicate that while non-complementary bundles typically weaken smart shopper feelings, framing the add-on as a free gift can neutralize this disadvantage, providing empirical support for Hypothesis 3.

### 4.5. Moderated Mediation Analysis

To test whether the effect of bundle complementarity on purchase intention was moderated by promotion framing and mediated by smart shopper feelings, we conducted a moderated mediation analysis using PROCESS (SPSS, v5.0) Model 7 with 5000 bootstrap resamples (Hayes, 2022). The results indicated significant moderated mediation (Index = −0.59, SE = 0.26; 95% CI [−1.11, −0.10]). This finding demonstrates that the indirect effect of bundle complementarity on purchase intention through smart shopper feelings is contingent upon the type of promotion framing.

Specifically, the mediation effect of bundle complementarity on purchase intention through smart shopper feelings was significant in the discount promotion condition (B = −0.82, SE = 0.22; 95% CI [−1.25, −0.39]), showing that when bundles were framed as a discount, the lack of complementarity lowered smart shopper feelings, which in turn reduced purchase intention. In contrast, the mediation effect was not significant in the free gift promotion condition (B = −0.12, SE = 0.22; 95% CI [−0.55, 0.31]), indicating that the free gift frame attenuated the negative pathway from non-complementarity to purchase intention.

**Figure 3:**
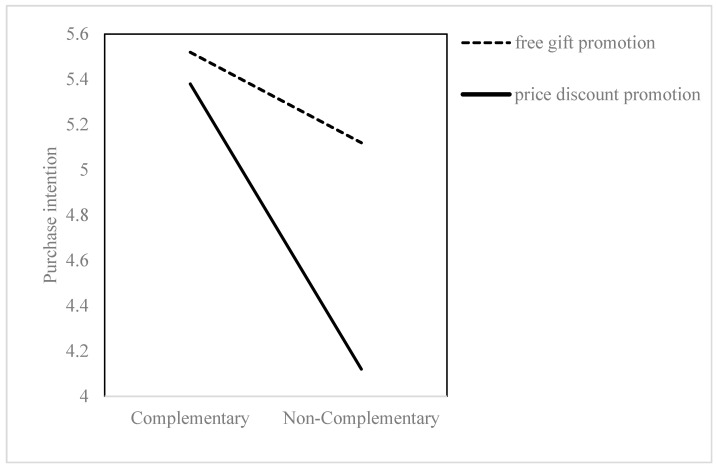
Promotion framing moderates the effect of bundle complementarity on purchase intention.

As illustrated in Figure 3, purchase intention followed a similar pattern to smart shopper feelings (Figure 2): they were significantly lower for non-complementary bundles under the discount frame, but this negative effect was neutralized under the free gift frame, resulting in purchase intentions for non-complementary bundles being comparable to those for complementary bundles. Together, these results provide robust evidence for Hypotheses 3 and 4, confirming that promotion framing moderates the mediating role of smart shopper feelings in the relationship between bundle complementarity and purchase intention.

## 5. Discussion

### 5.1. General Discussion

This research sought to resolve a puzzle in consumer psychology and marketing: how can firms effectively market non-complementary product bundles? Our findings reveal that the answer lies not in the bundle’s contents alone, but critically, in its promotional framing. We demonstrate that while a standard price discount accentuates the disadvantage of incongruent bundles, framing the ancillary item as a “free gift” neutralizes this disadvantage. In particular, the free gift frame prevented the decline in smart shopper feelings typically associated with non-complementary bundles, thereby sustaining purchase intentions at levels comparable to complementary bundles.

The findings from our research first confirmed the established principle that, all else being equal, consumers prefer the logical coherence of complementary bundles (H1), driven by the greater sense of being a smart shopper that these bundles evoke (H2). However, this baseline effect was moderated by the introduction of a “free gift” frame. When framed as a price discount, non-complementary bundles weakened consumers’ smart shopper feelings, reducing purchase intentions. In contrast, when framed as a free gift, this negative effect was attenuated: consumers experienced smart shopper feelings at levels similar to those reported in the complementary condition, which in turn sustained their purchase intentions (H3 and H4).

### 5.2. Theoretical Implications

This study offers several contributions to behavioral science theory. First, it contributes to bundling theory by identifying promotional framing as a critical boundary condition for the long-held principle of complementarity. Our findings suggest that the strategic importance of product fit is not absolute but can be significantly attenuated when a bundle is framed as containing a “free gift.” This perspective encourages a shift in theoretical focus—moving beyond an exclusive emphasis on what products to bundle, to include the crucial question of how the bundle’s value is psychologically communicated.

Second, the research advances the promotion framing literature by demonstrating the robustness of the “free” heuristic in a complex evaluation context. The findings indicate that the affective appeal of a “free” item is potent enough to counteract the cognitive dissonance that may arise from evaluating an incongruous, multi-item package. This offers a nuanced perspective on the “freebie devaluation” debate (Raghubir, 2004), suggesting that in cognitively demanding evaluations, the simple, positive “free” cue may dominate a more deliberative assessment of an item’s standalone value.

Finally, by empirically validating “smart shopper feelings” as the key mediating mechanism, this work highlights the integral role of self-conscious, affective responses in what is often modeled as a purely cognitive or economic decision. This research establishes these feelings as a key psychological state that drives consumer choice in bundle evaluations, thereby offering a richer, more psychologically grounded framework for future research into consumer decision-making.

### 5.3. Practical Implications

The findings present a significant strategic opportunity for marketers across various industries. Managers should feel empowered to move beyond obvious, traditional pairings and experiment with creative, non-complementary bundles. This strategy opens a new landscape for differentiation by forging unique partnerships that can create memorable and exclusive consumer experiences. The success of such a strategy, however, is not guaranteed by the partnership alone; it is critically dependent on the psychological framing of the offer.

This research highlights a key principle for promoting these creative packages: the ancillary benefit is more effective when framed as a “free gift” rather than a price discount. For example, in the experience economy, a promotion for a hotel and a museum ticket should not be marketed as “Stay & Museum Package: 20% Off!” but rather as “Book Your Stay, Get a Museum Ticket FREE!”. Our findings demonstrate this simple shift in wording can neutralize the negative impact of low complementarity by sustaining consumers’ smart shopper feelings, which are otherwise diminished under a discount frame. By preventing the decline in perceived value, the free gift framing enables non-complementary bundles to be evaluated as favorably as complementary ones, even when the final price to the consumer is identical.

### 5.4. Limitations and Future Research

While this study provides valuable insights, its limitations offer clear directions for future research.

First, our study was conducted using an experiential service bundle within a U.S. cultural context. Future research should test the generalizability of these effects across different domains, such as for tangible consumer goods, digital subscriptions, or B2B services. Furthermore, cross-cultural research could explore how the “smart shopper” construct and the appeal of “free” offers manifest in collectivistic versus individualistic cultures.

Second, the study relied on purchase intentions in a hypothetical scenario. While our stimuli were realistic, future research would benefit from field experiments that measure actual consumer behavior. A/B testing this framing strategy on a live e-commerce platform would provide invaluable data on real-world conversion rates and revenue impact.

Third, although we carefully controlled the objective value of the bundles across conditions, two alternative explanations warrant consideration. First, participants may have perceived the free gift frame as implying greater monetary savings, even though the total value ($90) and out-of-pocket cost ($70) were identical across conditions. Because we did not directly measure perceived savings, future research should incorporate such measures to more conclusively rule out this explanation. Second, the use of lunch as the complementary item may have been construed as a necessity rather than an optional add-on, potentially inflating its perceived value. Future studies could address this concern by employing discretionary complementary items of equal value (e.g., a gift shop voucher) to further test the robustness of our findings.

Fourth, our investigation was limited to a specific bundle structure where a smaller item was added to a larger one. Future research could expand upon our work by exploring bundles with different characteristics. For instance, studies could examine brand collaborations featuring products of comparable value (e.g., the Stanley and Lululemon collaboration).

Finally, future research could explore the long-term effects of this strategy. Does the “free gift” frame impact post-purchase satisfaction or brand loyalty? Does a consumer who received a “free” item subsequently devalue that item or the associated brand? Answering these questions would provide a more complete understanding of the strategic and psychological implications of using “free” as a promotional tool.

## Data Availability

Data can be obtained upon request from the corresponding author.

## Note

1 While some research has linked smart shopper feelings to the uniqueness or rarity of an offer—suggesting that consumers may feel savvy when obtaining something unusual or distinctive—our conceptualization follows a different line of work. Specifically, we emphasize justification and perceived coherence as the primary triggers of smart shopper feelings (Darke & Chung, 2005; Schindler, 1992; Zhang & Mick, 2019). From this standpoint, complementary bundles are more likely to evoke these feelings. We acknowledge, however, that rarity-based mechanisms may also play a role in other contexts, which represents a valuable direction for future research.

## Appendix A. Experimental Stimuli: Promotional Posters

All participants first read a scenario: “Please imagine that you are about to book a cruise for this upcoming Saturday. There’s a special promotion offering a cruise ticket and a lunch experience together for just $70.” They then viewed the poster of their assigned condition.
